# Wildlife usage indicates increased similarity between reclaimed upland habitat and mature boreal forest in the Athabasca Oil Sands Region of Alberta, Canada

**DOI:** 10.1371/journal.pone.0217556

**Published:** 2019-06-04

**Authors:** Virgil C. Hawkes, Travis G. Gerwing

**Affiliations:** 1 LGL Limited Environmental Research Associates, Sidney, British Columbia, Canada; 2 Department of Biology, University of Victoria, Victoria, British Columbia, Canada; 3 Ecosystem Science and Management Program, University of Northern British Columbia, Prince George, British Columbia, Canada; Trent University, CANADA

## Abstract

While there is no denying that oil sands development in the Athabasca Oil Sands Region (AOSR) has large impacts upon the habitat it disturbs, developers are legally required to return this land to “an equivalent land capability.” While still early in the process of reclamation, land undergoing reclamation offers an opportunity to study factors influencing reclamation success, as well as how reclaimed ecosystems function. As such, an Early Successional Wildlife Dynamics (ESWD) program was created to study how wildlife return to and use reclaimed upland boreal habitat in the AOSR. Wildlife data comprising 182 taxa of mammals, birds, and amphibians, collected between 2011 and 2017 and from five oil sands leases, were compared from multiple habitat types (burned [BRN], cleared [CLR], compensation lakes [COMP], logged [LOG], mature forest [MF], and reclaimed sites [REC]). Overall, similarity of wildlife communities in REC and MF plots varied greatly, even at 33 years since reclamation (31–62% with an average of 52%). However, an average community similarity of 52% so early in the successional process suggests that current reclamation efforts are progressing towards increased similarity compared to mature forest plots. Conversely, our data suggest that REC plots are recovering differently than plots impacted by natural (BRN) or other anthropogenic disturbances (LOG), which is likely due to differences associated with soil reconstruction and development on reclaimed plots. Regardless of the developmental trajectory of reclaimed habitats, progression towards increased wildlife community similarity at REC and MF plots is apparent in our data. While there is no expectation that reclaimed upland habitats will resemble or function identically to naturally occurring boreal forest, the degree of similarity observed in our study suggests that comparable ecological functionality is possible, increasing the probability that oil sands operators will be able to fulfill their regulatory requirements and duty to reclaim regarding wildlife and wildlife habitat.

## Introduction

Located in the northeastern portion of the Canadian province of Alberta, the Athabasca Oil Sands Region (AOSR) has received national and international attention regarding the environmental costs of large-scale resource extraction [1, 2]. Of the ~142,200 km^2^ of land in the boreal forest that comprise the Alberta oil sands deposits, 93,000 km^2^ occur in the AOSR, where ~4,800 km^2^ are part of the surface mineable area available for oil and gas development [1]. As of December 2017, ~895 km^2^ of the 4,800 km^2^ has been cleared or disturbed [3], all of which must be reclaimed [4]. Like most intensive resource extraction initiatives, the development of the Athabasca Oil Sands results in large-scale anthropogenic disturbances [1, 2] and poses substantive challenges for conservation, land management, and habitat reclamation [5]. Given the duration of operations associated with most oil sands mines in the AOSR (many exceed 50 years at current planned production rates), long-term disturbance to wildlife and their habitat is unavoidable. The primary agent of disturbance is habitat loss and isolation [6], leading to area and edge effects that can impact biodiversity [7] and alter the abundance and composition of flora and fauna near disturbed sites [8–11]. These impacts will have variable effects on wildlife species: some will adapt to a more fragmented environment while others will not and may become at risk of extirpation within the project area. However, oil sands developers are legally required to return disturbed land to “an equivalent land capability [1, 4],” defined as the ability of the land to support similar, but not necessarily identical land uses that existed prior to disturbance [3, 12].

To determine whether wildlife is returning to and using reclaimed habitats in the AOSR, long-term monitoring of those landscapes, as well as suitable natural analogues are required. Long-term monitoring provides an opportunity to study how reclaimed ecosystems develop over time and contributes to an increased understanding of how reclaimed ecosystems function [13]. Wildlife use of reclaimed habitats in the AOSR has only recently received study (e.g.,[5, 14]). Baseline studies have been conducted, mainly to support the Environmental Assessment process, and while monitoring does occur, there are currently few quantitative data from which an assessment of reclamation effectiveness can be made. Lack of study is mainly due to the early stage at which most operators are at in their reclamation process. Substantial areas will be reclaimed over the next 25 years [1, 15], but the majority (~68%) of all reclamation to occur over the lifetime of oil sands mines will occur after 2035 [16]. This apparent delay in reclaiming habitat is related to the time frame over which mines will be operational (decades) as well as the spatial scale of the mines (> 100km^2^). As such, little is known about the ability of reclaimed habitats to provide habitat for wildlife. Moreover, mining related land reclamation at the spatiotemporal scale mandated in the AOSR is relatively novel, with no such efforts known from anywhere in the world. To address these shortcomings, an early successional wildlife dynamics (ESWD) program was developed and implemented on multiple oil sands leases in the AOSR. This program is tasked with understanding how wildlife is returning to and using reclaimed habitats, as well as assessing the point in time when reclaimed upland habitats provide functional wildlife habitat that is similar to undisturbed boreal forest.

This paper describes the development and implementation of the ESWD program, which studies wildlife use of reclaimed upland boreal habitat, not only to determine reclamation progress as contrasted with undisturbed mature boreal forest, but also to provide real time data to enable adaptive management. Adaptive management is a key component of an integrated monitoring program that aims to assess reclamation effectiveness [13, 17]. The ESWD program not only assesses how wildlife is returning to and using reclaimed upland habitat, but it also assesses wildlife usage of analogues (burned, logged, and cleared sites) of similar age. Such *a priori* contrasts allow us to determine if habitat in the process of being reclaimed is progressing along developmental trajectories similar to habitats not disturbed by mining. Although the total area of disturbed land that has been reclaimed is currently small relative to the impact (~77 km^2^ vs. 895 km^2^), studying patterns of wildlife colonization and occupancy at the outset of reclamation should provide data necessary to understand developmental trajectories of reclaimed habitats. Furthermore, study of reclaimed habitats offers a unique opportunity to assess the effectiveness of reclamation strategies that have been implemented before most habitat reclamation occurs [18]. This paper also presents an initial evaluation of the effectiveness of the ESWD program. This assessment is conducted at an ecosystem level (taxa presence/absence) and does not focus upon taxa-specific trends to highlight broad reclamation progression. Forthcoming manuscripts will focus upon individual taxa (song birds, small mammals, bats, arthropods, etc.) and provide in-depth analyses of their response to reclamation measures.

By necessity, the description of the ESWD program requires a brief overview of the AOSR; however, this is not the focus of this paper, and readers interested in further detail can refer to Gosselin, Hrudey [1] or Audet, Pinno [19]. Instead, our paper focuses on the theory behind the development of the ESWD program, the utility of multi-taxa studies to assess reclamation success, as well as key practical realties. Lastly, the effectiveness of the ESWD program to assess upland reclamation success relative to developmental trajectories of wildlife habitat is examined. It is important to note that the ESWD program currently focuses only on assessing the efficacy of upland reclamation, and we do not explore reclamation of wetland habitats or tailings ponds. Interested readers can refer to Allen [20], Johnson and Miyanishi [6], Gosselin, Hrudey [1], and Rooney, Bayley [15] for discussions surrounding wetland restoration in the AOSR.

### Study area: Athabasca Oil Sands Region

**Fig 1:**
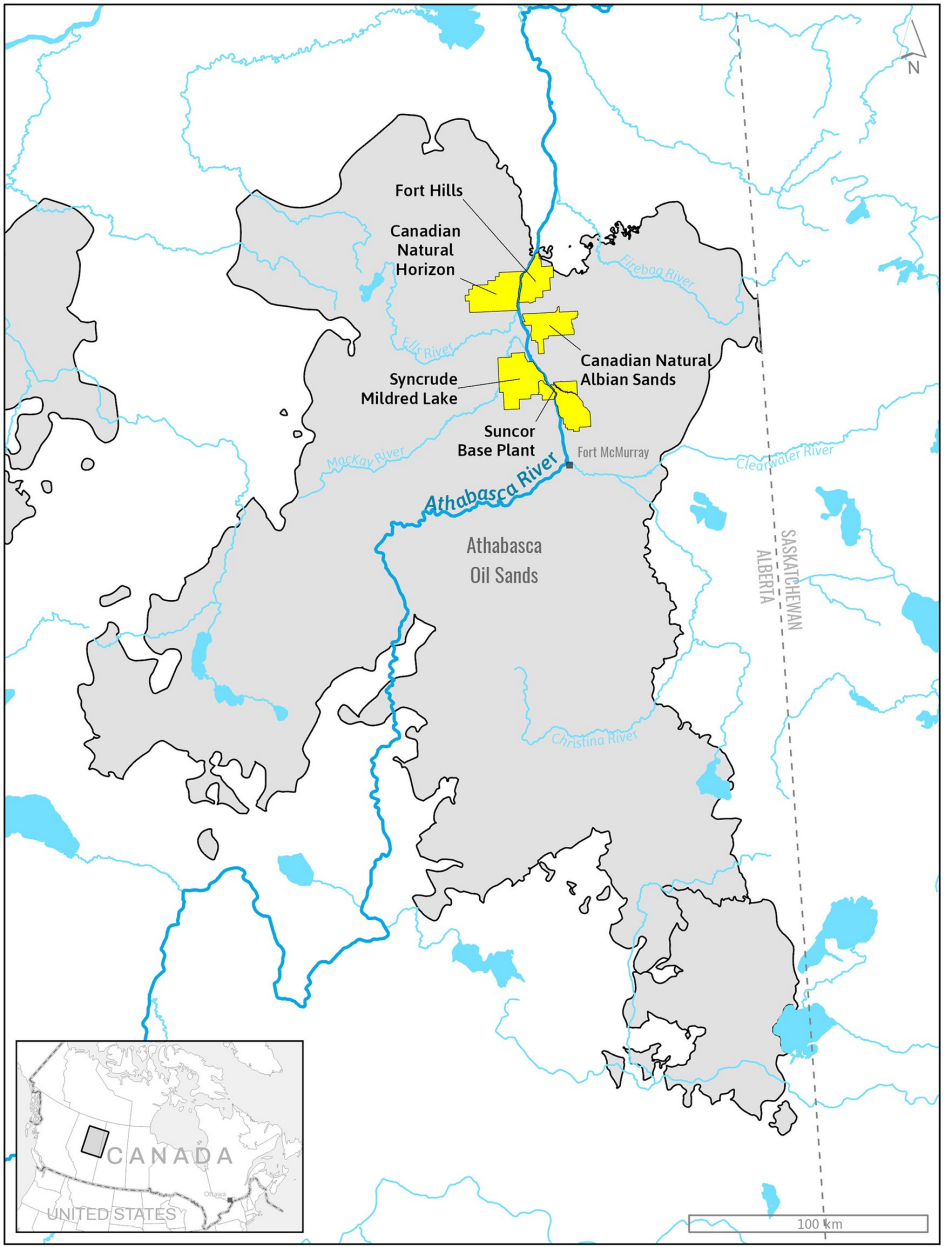
Map of the Athabasca Oil Sands Region created using ArcGIS Pro 2.2 and the oil sands leases monitored by the early successional wildlife dynamic program.

The AOSR (Fig 1), located ~440km northeast of Edmonton, Alberta, Canada, is the largest of three oil sands deposits in Alberta, and covers ~93,000km^2^ surrounding the community of Fort McMurry [1, 21]. This area lies in the North America Boreal Plain, a relatively flat region (400-800m above sea level) that until 10,000–12,000 years ago, was covered by the Laurentide ice sheet [6]. Oil sands surface mineable deposits are contained within surface glacial deposits at depths of 30 to 200 m, and are composed of loamy till, gravel, and sand [6]. Along with logging and oil sands development, fire and insect pests continue to be dominant sources of disturbance on this landscape [6, 22].

Within the AOSR, the ESWD program has been implemented on five open pit mine leases (Fig 1): Canadian Natural’s Horizon Oil Sands, Shell Albian Sands (now Canadian Natural Albian Sands), Suncor Energy’s Oil Sands Base, and Fort Hills Oil Sands Project. Some data used in this report were obtained under contract to the Cumulative Environmental Management Association (CEMA) in 2010, 2011, and 2012. This includes comparable data collected on Syncrude’s Mildred Lake Lease. These data are used with permission. Sampling for the ESWD program occurs on six different site types: (1) reclaimed (REC); (2) reclaimed habitat adjacent to compensation lake (COMP); (3) mature forest (MF); (4) cleared habitats (CLR); (5) logged (LOG); and (6) burned (BRN). Upland reclamation intends to recreate an upland boreal forest ecosite type (as per Beckingham and Archibald [23]) on reconstructed soils that were vegetated with native herbs, forbs, shrubs, and trees to be consistent with species in the naturally occurring and surrounding boreal forest. Reclaimed habitats sampled for the ESWD program ranged in age from 2 to 33 years post-reclamation (Table 1) and were created in areas previously disturbed through a combination of mining and clearing activities. Habitats adjacent to compensation lakes were also sampled. These habitats represent reclaimed habitats as they were disturbed in manners similar to REC plots during creation of compensation lakes. However, COMP plots are treated as a separate category of reclamation as current data suggest that certain groups of wildlife (e.g., songbirds) vary around compensation lakes [24, 25]. Relatively intact (i.e., few cut lines, roads or other human-associated disturbance) mixedwood leading to pure coniferous and deciduous mature forest sites at least 10 ha in size and representing the desired endpoint of upland reclamation were selected for monitoring. The size difference between mature forest and reclaimed sites is to enable sampling of mature forests in the absence of edge effects. Further, land that was cleared of all vegetation (CLR) and left to regenerate on its own, with vegetation returning via ingress and natural succession, was also sampled. Most of the cleared sites are < 20 years old, placing them in the same age class as upland reclamation sites. Lastly, juvenile stands recovering from logging (LOG) and stand-replacing fire (BRN) represent the best available analogues to compare the developmental trajectories of wildlife habitat and use relative to upland reclamation [22, 26]. LOG plots vary from CLR plots in that all vegetation was removed from CLR plots, while in LOG plots only harvestable timber was removed. Although the site-level disturbance associated with logging and fire is different from upland reclamation, the approach to upland reclamation emulates the approach used to reforest sites post-logging. The age since stand-replacing fire is similar to the time since reclamation for many reclaimed sites currently included in the ESWD, providing a suitable comparison regarding the return to and use of reclaimed habitats relative to naturally regenerating stands following fire [22, 26]. Similarly, data from logged sites of the same age can function to assess whether upland reclamation promotes the establishment and development of vegetation structure that is similar to the communities that develop following logging [26].

**Table 1:** Summary of plots sampled as part of the Early Successional Wildlife Dynamics Program in the Athabasca Oilsands Region.

Treatment	N	Age	Number of Plots
BRN	12	2	2
		3	2
		4	2
		5	3
		6	3
CLR	5	5	1
		6	1
		8	1
		11	2
COMP	20	2	1
		3	2
		3	2
		4	5
		5	4
		6	4
		7	2
LOG	7	4	1
		5	3
		6	3
MF	20	NA	7
REC	61	1	2
		2	5
		3	4
		4	9
		5	6
		6	9
		7	3
		8	1
		9	3
		10	4
		11	1
		12	2
		14	1
		16	1
		18	5
		19	2
		20	1
		26	1
		33	1

Age relates to the number of years have passed since reclamation or cessation of disturbance as of 2017. BRN: Burned; CLR: Cleared; COMP: Compensation Lake Forest; LOG: Logged; MF: Mature Forest; REC: Reclaimed. N = number of year X plot combinations, sampled between 2011 and 2017, that were included in our assessment of the ESWD. Year X plot combinations were included only if they contained quantifiable data from all data categories (small mammal trapping grid, bird point count stations, as well as wildlife cameras).

### Early Successional Wildlife Dynamics—Monitoring framework

#### Goals of the ESWD

The primary goals of the ESWD program include: (1) development of wildlife data profiles for all site types to determine if developmental trajectories of reclaimed habitats align with naturally regenerating juvenile stands, and at what point reclaimed habitats achieves the desired endpoint; and (2) provision of guidance to reclamation practitioners regarding wildlife use of reclaimed habitats that could be incorporated into future reclamation plans.

#### Reference sites and restoration trajectories

To assess reclamation success and/or progress, data from reclaimed habitat must be compared to undisturbed reference habitat. However, the impact of variable and changing ecosystems must be accounted for when selecting reference areas. Ecosystems are neither static nor homogenous, often capable of existing in multiple stable states that naturally vary over space and time [6, 27, 28]. As such, it is difficult to set achievable reclamation goals or benchmarks based upon one set of historic pre-disturbance conditions, as they may not accurately reflect current conditions of undisturbed sites, or historic conditions may represent only one of a suite of potential states [6, 15, 27, 28]. Moreover, successional pathways that lead to historic conditions may no longer be possible under current environmental conditions [6, 27, 28]. Failure to account for a changing ecosystem and multiple stable states has led to the failure of reclamation projects in the past [1, 28]. Therefore, it is better to attempt to create ecosystems with specific biotic and abiotic processes/services, while using historical information as a useful guideline [6, 28]. More specific to the AOSR, Johnson and Miyanishi [6] argue that given the area (≥ 100km^2^ for each mine) that will eventually be impacted, reclamation in the oil sands is not really reclamation, but engineering of an entirely new ecosystem. As such, reclamation to identical pre-disturbance conditions is likely impossible [1, 6, 28]. As such, reference boreal forest sites must represent the natural variety of this target ecosystem, acknowledging that multiple stable end points are possible within reference communities [28]. Further, monitoring the successional dynamics at study sites impacted by non-mining related disturbances such as logging, clearing, or forest fires [1, 26, 28] will illuminate if reclaimed sites are developing along successional trajectories present in the ecosystem.

#### Early Successional Wildlife Dynamics and adaptive management

As discussed above, adaptive management will be critical to the EWSD program as revegetation prescriptions associated with each reclaimed area have not always been adequately described, and management activities as well as their success/failures have not always been tracked. Finally, the rates of natural plant ingress have not been well documented, particularly for non-tree species. These confounding factors need to be controlled for, which is why establishing an adaptive management component will allow for modification of the ESWD program based on real-time results.

#### Wildlife monitoring: Selection of indicator species

The ESWD program focuses on taxonomic groups that are considered indicative of reclamation success. Indicator species were determined partially through pilot programs conducted in the AOSR, and from the results of similar programs implemented between 2012 and 2015 on Canadian Natural’s Horizon Oil Sands, Suncor Energy’s Oil Sands Base, and Shell Albian Sands [29]. Even with pre-existing data, the selection of indicators can be challenging [30–32] and should be guided by species sensitivity to management practices, ease of data collection, and usefulness of the information to address management activity [31–33]. Potential indicators may include habitat attributes, keystone species, species at risk, species associated with specific habitat requirements, or species that can be monitored easily [31, 33, 34]. Critically, their selection should also be appropriate to the spatial scale of the applied management activity [35, 36]. Selection of indicators must also take into consideration factors external to the monitoring program, such as inter and intra-specific competition, predation, disease, and seasonal variation in temperature and precipitation rates [31–34, 37].

Due to the impracticality of monitoring all species of wildlife occurring in the AOSR, fourteen focal taxa (Table 2) were selected by the Cumulative Environmental Management Association (CEMA) to represent wildlife communities considered to be of ecological or socio-economic importance in the region [12, 38]. Of these fourteen taxa identified as potential indicators, focal taxa were selected for the ESWD and include small mammals, bats, songbirds, amphibians, terrestrial arthropods and winter-active animals. Some taxa such as arthropods not identified by CEMA were included in the ESWD as inclusion of these taxa in the monitoring framework offers greater insight into recovery and functionality of boreal habitat. All taxa were selected based on several criteria, including their use as key indicators in environmental impact assessments. Bat species were monitored relative to habitat type as certain bat species (*Myotis lucifugus* and *M*. *septentrionalis*) have current provincial (May be at Risk) or federal (Endangered) conservation designation. Human impacts to the landscape resulting from bitumen extraction can significantly affect populations of forest-dwelling bats, diminishing the ecological roles they provide [39–41]. Individually each of these taxonomic groups–birds [42, 43], bats [40, 44], mammals [45, 46], and insects [35, 47]–are good indicators of anthropogenic disturbances, and functionality of an ecosystem. However, when monitored together, these species allow us to holistically assess reclamation progress [1, 6, 26].

**Table 2:** Cumulative Environmental Management Association (CEMA) Sustainable Ecosystem Work Group goals and indicators for wildlife, including habitat reclamation.

Goal	Indicator	Rationale
Sustain viable & healthy populations of wildlife	All species	All wildlife are interconnected
Protect & sustain unique, threatened, endangered & other species of concern	Canadian Toad	‘At risk’ designation (red list in Alberta)
	Woodland Caribou	‘Threatened’ designation (blue list & COSEWIC)
Sustain wildlife species with an important ecological role	Lynx / Snowshoe Hare	Key mammal predator/prey dynamic in region
	Pileated Woodpecker	Creates habitat for cavity-nesting birds & mammals
	American Beaver	Engineers habitat & thereby manipulates distribution of water & soil nutrients
Sustain wildlife species that are habitat specialists	Old growth forest bird community	Require structural elements found in old forests (>100 y)
	Boreal Owl	Require structural elements found in old forests (>100 y)
	North American River Otter	Require moving water habitats (streams, rivers)
Sustain species that are important for cultural, spiritual, medicinal & ceremonial purposes	American Black Bear	A powerful spirit animal important to Aboriginal people for all purposes listed
Sustain wildlife populations for subsistence, commercial and/or recreational hunting, fishing & trapping	Moose	Remains a staple country food, cultural keystone species
	Common Muskrat	Foundation of traditional trap-lines
	Fisher / Red-Backed Vole	Important fur species & its key prey base
	Ruffed Grouse	Valued upland game bird
Sustain wildlife populations for recreational non-consumptive use	Mixed wood forest bird community	Aesthetic value for bird-watchers, photographers, hikers, etc.

#### Wildlife sampling

Sampling units were stratified [48, 49] across the AOSR by lease, and within each lease by habitat type. In general, if a reclamation area was ≥ 5 ha, non-linear, within 500 m of existing reclamation or natural areas, and was reclaimed using methods that are likely to be used in the future, the site was selected for monitoring. Within a habitat type, a plot was established in the approximate centre of that habitat patch, and specific sampling locations for birds and mammals were established as randomly as possible. However, accessibility and availability of appropriate habitat within a plot constrained sampling location. While habitat patch size varied based upon availability, sampling areas (i.e., size, shape, experimental unit) within each plot were kept consistent to enable contrasts between plots and treatments. Annual wildlife sampling occurred during all seasons, with data collected during systematic surveys augmented by data collected via remote-sensing equipment [wildlife cameras and autonomous recording units (ARUs)]. Sampling effort and area was kept as consistent as possible between years. Survey methods remained consistent among observers, programs, years, and locations, ensuring comparability of results. Small mammal live-trapping mark-recapture surveys were used to document species composition and density of small mammals across each sampling area, specifically providing data on the focal species, Southern Red-backed Vole (*Myodes gapperi*). Baited with peanut butter, oats, apples, and carrots, Sherman (H.B. Sherman, Inc.) and Little Critter (Longworth-style) traps were placed in a 7x7 grid covering 100m^2^ and were checked twice-daily for up to 10 days, but less if grids were disturbed by bears. Songbird point count surveys were used to document mixedwood and old growth forest bird communities. Six experienced observers completed breeding bird surveys at 207, 6-miute point count stations as per Ralph, Sauer [50]. Bat species and activity were assessed via ARUs (Wildlife Acoustics, Inc. Song Meter SM2+BAT and SM4) outfitted with ultrasonic microphones. Passive wildlife detection via remotely triggered cameras (Reconyx PC800) collected data on numerous focal taxa. Presence of amphibians were assessed opportunistically (visually and auditorily) while conducting all other surveys in upland boreal habitat.

## Evaluation of the ESWD program and assessment of Early Restoration Progress

Permits for this work were provided by the Alberta Environment and Parks, Policy and Planning Division, Fish and Wildlife Policy Branch.

## Materials and methods

### Statistical analyses

Data were available from five oil sands leases (Fig 1) and multiple habitat types (MF, REC, CLR, LOG, BRN, and COMP) in the AOSR around Ft. McMurry, Alberta (476878.82 m E, and 6287053.39 m N). Not all taxa observations from all methods of data collection (small mammal trapping grids, bird point counts, wildlife cameras, ARUs, and incidental wildlife observations) were appropriate for quantitative analysis of data. Specifically, only plots for which data from small mammal live-trapping grids, bird point counts, and remotely triggered wildlife cameras were available were used in quantitative analyses (Table 1). Bats were uniformly present at all plots (but varied in activity) and were therefore removed from analyses to focus upon taxa that varied in their presence/absence. This resulted in a total of 125 applicable plot/year combinations (from a total of 282) consisting of a taxa matrix of 182 taxa, sampled from 2011–2017. Sample size varied by treatment type (BRN: 12 plots; CLR: 5; COMP: 20; LOG: 7; MF: 20; and REC: 61; Table 1). Individuals not identifiable to species were retained in the analysis to better represent the taxonomic diversity observed on site. Inclusion or exclusion of these taxa did not influence the reported trends. All subsequent analyses were conducted upon a taxa matrix of presence/absence data. Presence/absence data equalizes the influence of rare and common taxa on downstream analyses, and enables us to examine taxa composition more easily, better elucidating species recovery and how this contributes to differences between habitat types over time [51–55]. Future investigations into this dataset will analyze habitat usage as indicated by density/abundance of all species, as well as in-depth analysis into species specific responses (e.g., birds, bats, mammals, and insects).

Data analyses were performed in PRIMER with the PERMANOVA add-on [51–55]. Our first objective was to compare REC sites to MF sites. In PRIMER the similarity percentages (SIMPER; [51–55]) routine was used to contrast REC plots (n = 61) to every MF plot (n = 20), determining the percent similarity of the wildlife communities (presence/absence; Bray-Curtis Similarity). Each REC plot was contrasted using SIMPER to every MF plot (n = 1160 contrasts). Percent similarities values were then plotted against the number of years since a plot was reclaimed to determine if REC plots are becoming more similar to MF plots with time. Time since reclamation (age) was determined by subtracting the year a plot was either planted/seeded (LOG, REC, and COMP) or the year disturbance ceased (BRN and CLR) from the sampling year. This generated an age range of 0–33 years since reclamation for all non-mature forest habitat types. Relationship between similarity and age was assessed with regression models [56]. A cubic function was fit to the data. The second goal of this analyses was to compare recovery trajectories of all habitat types. Non-metric multidimensional scaling plots (nMDS) with 100 restarts [51] were used to compare wildlife communities at all treatments over time. The response variable for all nMDS plots were resemblance matrices constructed from wildlife community data. The resemblance matrix was calculated using Bray-Curtis coefficients [51]. All MDS graphs had a stress ≤0.2, and so were considered good 2-dimensonal representation of higher dimensional trends [51].

## Results

A total of 182 taxa of wildlife (S1 and S2 Tables) were documented from all sampled oil sands leases. Overall more taxa were detected on REC habitats (n = 133) and those habitats were also associated with the largest number of unique taxa (n = 25). COMP habitats were associated with 127 taxa, of which 20 were unique. On BRN sites 72 taxa were observed and only 2 were unique. LOG and CLR habitats were the least with 54 and 55 taxa, respectively, and neither treatment was associated with any unique taxa of wildlife. Finally, 95 taxa of wildlife were observed in MF reference sites, 15 of which were unique. Each of the taxa associated with a given treatment was expected based on known patterns of wildlife habitat use, occurrence and distribution. This includes those taxa unique to a given habitat type (S1 and S2 Tables).

**Fig 2:**
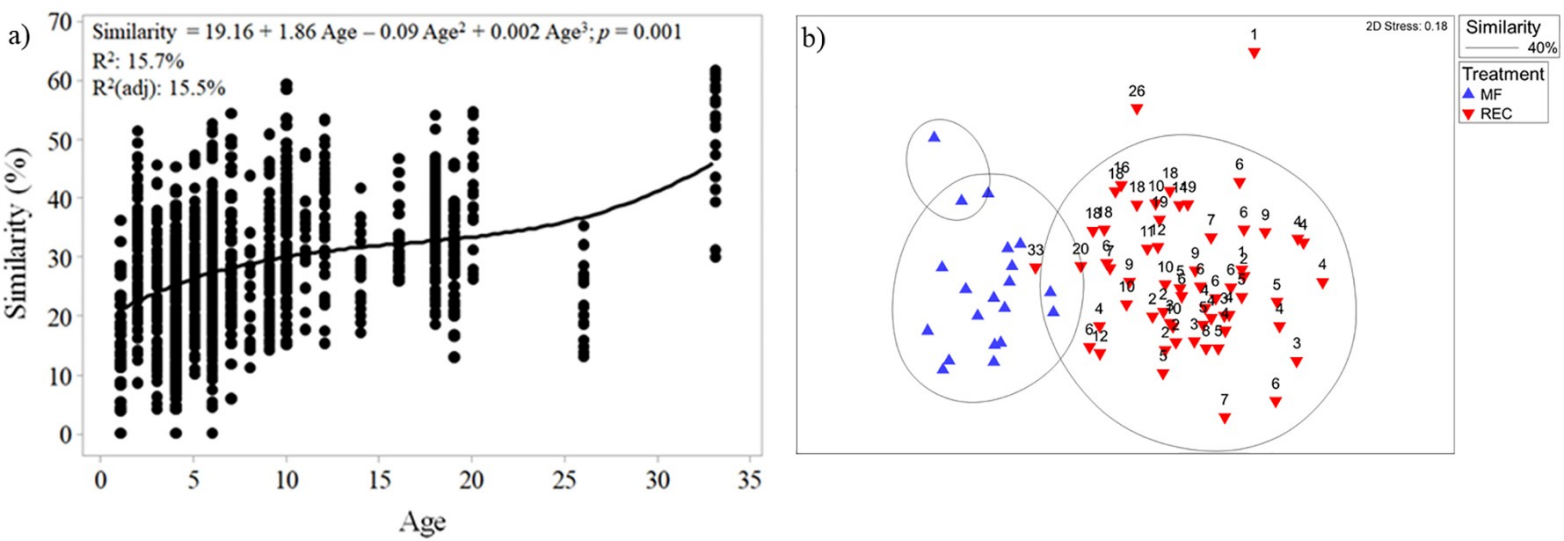
Relationship between wildlife communities in mature forest and reclaimed plots in the Athabasca Oil Sands Region. a) community similarity (%) between reclamation and mature forest plots, against years since a plot was reclaimed. b) non-metric multidimensional scaling plot (nMDS) showing the relationship between mature forest (MF) and reclamation (REC) plot wildlife communities.

**Fig 3:**
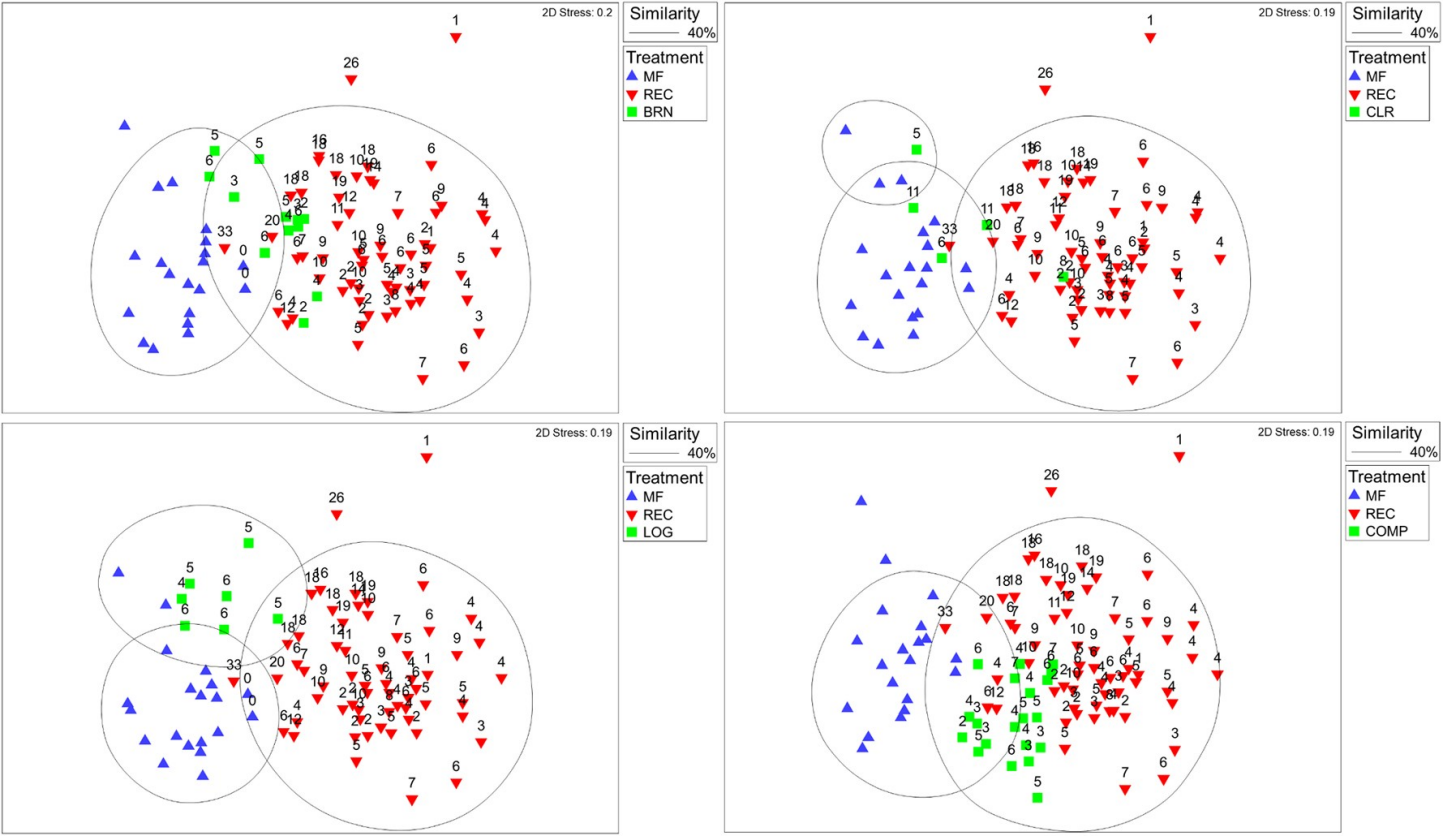
Non-metric multidimensional scaling plots (nMDS) showing relationships between wildlife communities in various reclamation treatments in the Athabasca Oil Sands Region (BRN: Burned; CLR: Cleared; COMP: Compensation Lake Forest; LOG: Logged; MF: Mature Forest; REC: Reclaimed). The number above each point is the number of years since the plot was reclaimed. Mature forest points are not presented with an age as they were not reclaimed.

Considerable variation was observed in the wildlife community across all spatial and temporal scales (Figs 2 and 3). In general, REC plots increased in similarity to MF plots over time, with wildlife communities at the oldest REC plots (33 years) starting to cluster with MF plots (Fig 2b). The cubic function (S3 Table) describing this relationship in Fig 2a is significant (*p* = 0.001), however, it only explains a small proportion of the observed variation (R^2^ = ~15%), emphasizing observed variation in wildlife communities. Further, there was considerable variation in similarity between REC and MF sites each year, with some REC plots resembling MF plots more or less. Even at 33 years of age, similarity of REC to MF plots varied from 31% to 62%, with an average of 52%. Burned plots (BRN; aged 2–6 years) did not group with MF plots nor with early REC plots, but with older REC plots aged 6–20 years (Fig 3). Cleared plots (CLR, aged 5,6,8, and 11–16 years) exhibited substantial variation (Fig 3). One CLR plot aged 11 years clustered with REC plots aged 2–10, while another CLR plot aged 11 years clustered with REC plots 18–20 years old. Conversely, three other CLR plots aged 5,6, and 11 clustered with MF plots. Logged plots (LOG) aged 4–6 years clustered with MF plots, and older REC plots aged 18–33 years (Fig 3). However, LOG plots appear to form a semi-distinct group between REC and MF plots. Finally, plots near compensation lakes (COMP) aged 2–7 years, clustered with REC plots aged 2–10 years (Fig 3).

## Discussion

To assess reclamation progress with regards to wildlife usage in habitats disturbed by mining activities in the AOSR, as well as to assess the early applicability of the ESWD program, wildlife communities were studied at various treatments (REC, BRN, LOG, COMP, and CLR), and at several times since reclamation (age; 1–33 years). Current developmental progress was assessed by comparing wildlife communities of REC to MF plots. Overall, wildlife communities (presence/absence) at REC plots increased in similarity to reference plots over time, supporting preliminary observations of bird community succession in the oil sands [5, 57]. Similarity of wildlife communities in REC and MF plots varied greatly, even at 33 years since reclamation (31–62% with an average of 52%). This suggests that even though reclaimed plots are over time starting to resemble mature boreal forest wildlife communities, variation still exists. Rowland, Prescott [26] observed a similar relationship regarding plant communities, with REC plots resembling vegetative conditions at MF sites more over time, but differences remained. Overall, 33 years post-disturbance the average similarity between wildlife communities in REC and MF plots was 52%. Such a high community similarity value is promising from a reclamation success perspective, as upland stands 33 years of age are still immature relative to mature boreal forest, and vegetative communities appear to stabilize 25 years following reclamation [58]. More generally, plant communities in the boreal forest start to resemble mature plots at ~50 years of age, with resemblance increasing at 60–100 years; however, succession can continue on these plots 250–300 years following disturbance, with few stands transitioning into old growth forest before natural disturbances such as fire resets succession [59–62]. That REC plots on average exhibit wildlife communities ~52% similar to MF reference plots only 33 years into the recovery process suggests that current upland reclamation efforts in the AOSR is progressing towards increased similarity when compared to mature forest plots. However, further assessments of habitat productivity and function are needed before reclamation success can be more fully assessed.

With regards to recovery trajectories, evidence suggests that some reclaimed habitat may be progressing along different successional trajectories than habitat disturbed by non-mining related activities (S4 Table). For instance, BRN plots (2–6 years old) did not cluster with REC plots of similar age, nor with MF plots. Instead they clustered with older REC plots, aged 6–20 year (Fig 3a). Similarly, LOG plots, aged 4–6 years, clustered with MF plots and older REC plots, those aged 18–33 years (Fig 3c). However, LOG plots do appear to be clustering together between MF and REC plots. These data suggest that REC plots are recovering differently than plots impacted by fire or logging. At the very least, REC plots appear to be progressing along the recovery trajectory slower than plots impacted by fire or logging. This trend is likely due to the nature of disturbance, as mining related disturbances not only remove above ground vegetation, but also have considerable impacts upon the soil. The reclaimed upland habitats sampled are constructed landforms with a manufactured soil layer that is overlain with forest floor mineral mix or Peat Mineral Mix which is the overburden (soil layer) that was removed after initial vegetation clearing of the mine lease. This material was either stockpiled for later use or used to reclaim habitats via direct placement [18, 22, 63, 64]. While fire and logging are both associated with the removal or damage of existing vegetation, soil is left more or less intact, aiding in vegetative and wildlife recovery. CLR plots represent an interesting source of disturbance, having had all their vegetation removed (not just harvestable timber), however, the soil is left mostly intact. Interestingly, younger CLR plots (5–11 years) clustered both with MF plots and with older REC plots, perhaps suggesting that CLR plots are also recovering more quickly than REC plots, likely due to minimal disturbance of the soil [18, 22, 63, 64]. COMP plots, on the other hand, cluster with REC plots of similar age (Fig 3d), potentially as COMP plots have had their soil characteristics reconstructed, therefore exhibiting a disturbance regime similar to that of REC plots. When taken together these data suggest that REC plots may be proceeding along a different successional trajectory than plots disturbed by fire or logging. More study is required to determine the end points of these recovery trajectories, as 33 years post disturbance is still early in boreal forest succession [59–62]. Therefore, more time and continued monitoring is required before we can determine if the outcome of REC plot recovery trajectories are greatly divergent from those of other types of disturbance (human and natural), or if wildlife communities in REC plots will continue to progress towards those of existing boreal forest.

### Future directions

Assessing the return to and use of reclaimed habitat by wildlife will continue under the ESWD program in the AOSR. One of the current data deficiencies is the low sample size of older REC plots. However, as more habitat is reclaimed, and already reclaimed plots progress through the recovery process [1, 16], more data will become available. Such data will help elucidate the recovery trajectories of all sampled habitat types, as well as offer insight into the functionality of restored habitats in general. Further, representative terrestrial arthropod data are now available and these powerful indicator species [22, 35] can be included in future analyses. Finally, further analyses of current ESWD data will include in-depth examinations of abundance and density trends of the wildlife community and individual species, as well as demographic data, offering further in-sight into reclamation success and trajectories.

### Conclusions

While there is no denying that oil sands developments have large impacts upon the landscape of the AOSR, developers are legally required to return disturbed land to “an equivalent land capability” [1, 4]. Even though it is still early in the process of reclamation, land currently undergoing reclamation offers a great opportunity to study factors influencing reclamation success, as well as how reclaimed ecosystems function. As such, the ESWD program studies wildlife use of reclaimed upland boreal habitat, not only to determine reclamation progress as contrasted with undisturbed mature boreal forest and natural analogs, but also to provide real time data to enable adaptive management. Overall, similarity of wildlife communities in REC and MF plots varied greatly, even at 33 years since reclamation (31–62% with an average of 52%). However, an average community similarity of 52% after 33 years is promising from a reclamation success perspective, as 33 years is early in the boreal successional process. These findings suggest that current reclamation efforts of upland boreal forest in the AOSR are progressing towards increased similarity compared to mature forest plots. Conversely, our data suggest that REC plots are recovering differently than plots impacted by natural (BRN) or other anthropogenic disturbances (LOG). Regardless of the developmental trajectory of reclaimed habitats (REC) relative to COMP, LOG, and CLR, a progression towards wildlife community similarity of REC to MF plots is apparent in our data. While there is no expectation that reclaimed upland habitats will resemble or function identically to naturally occurring boreal forest [1, 6, 27] the degree of similarity observed further suggests that similar functionality is possible, increasing the probability that oil sands operators will be able to fulfill their regulatory requirements and duty to reclaim regarding wildlife and wildlife habitat.

## Supporting information

S1 Table Presence/Absence of species observed in the Alberta Oil Sands Region as part of the Early Successional Wildlife Dynamics Program. BRN: Burned; CLR: Cleared; COMP: Compensation Lake Forest; LOG: Logged; MF: Mature Forest; REC: Reclaimed. Unidentified species were retained in the analysis to better represent the taxonomic diversity observed on site. Inclusion or exclusion of unidentified taxa did not influence the reported trends.
(DOCX)

S2 Table Raw data. (XLSX)

S3 Table Comparison of linear, quadradic, and cubic models comparing community similarity against time since reclamation (age) in the Athabasca Oil Sands Region. (DOCX)

S4 Table Similarities percentages (SIMPER) tables showing species contributions to differences between habitat types. (XLSX)

## References

[1] Gosselin P, Hrudey SE, Naeth MA, Plourde A, Therrien R, Van Der Kraak G, et al Environmental and health impacts of Canada’s oil sands industry. Royal Society of Canada, Ottawa, ON 438 p. 2010.

[2] Giesy JP, Anderson JC, Wiseman SB. Alberta oil sands development. Proceedings of the National Academy of Sciences. 2010;107(3):951–2. 10.1073/pnas.0912880107 PMC2824323 20080655

[3] AE. Alberta Energy. Oil Sands. http://www.energy.alberta.ca/OS/AOS/Pages/FAS.aspx. 2018.

[4] EPEA. Environmental Protection and Enhancement Act. http://www.qp.alberta.ca/documents/Acts/E12.pdf. 2009.

[5] Foster KR, Godwin CM, Pyle P, Saracco JF. Reclamation and habitat-disturbance effects on landbird abundance and productivity indices in the oil sands region of northeastern Alberta, Canada. Restoration Ecology. 2017;25(4):532–8.

[6] Johnson EA, Miyanishi K. Creating new landscapes and ecosystems: The Alberta Oilsands. Annals of the New York Academy of Sciences. 2008;1134(1):120–45. 18566092 10.1196/annals.1439.007

[7] Cristescu B, Stenhouse GB, Symbaluk M, Nielsen SE, Boyce MS. Wildlife habitat selection on landscapes with industrial disturbance. Environmental Conservation. 2016;43(4):327–36.

[8] Andren H. Effects of habitat fragmentation on birds and mammals in landscapes with different proportions of suitable habitat: A review. Oikos. 1994;71:355–66.

[9] Fahrig L. Effects of habitat fragmentation on biodiversity. Annual Review of Ecology, Evolution, and Systematics. 2003:487–515.

[10] Fahrig L. Relative effects of habitat loss and fragmentation on population extinction. The Journal of Wildlife Management. 1997;61:603–10.

[11] Schmiegelow FKA, Mönkkönen M. Habitat loss and fragmentation in dynamic landscapes: Avian perspectives from the boreal forest. Ecological Applications. 2002;12(2):375–89.

[12] AENV. Alberta Environment. Guidelines for reclamation to forest vegetation in the Athabasca Oil Sands Region, 2nd Edition. Prepared by the Terrestrial Subgroup of the Reclamation Working Group of the Cumulative Environmental Management Association, Fort McMurray, AB. December 2009. 2010.

[13] Hawkes VC, Donald G. Reclamation monitoring in the Athabasca oil sands region of Canada using a long-term plot network. Pages 717–728 In A.B. Fourie and M. Tibbet (eds.) Mine Closure 2012. Proceedings of the seventh international conference on mine closures. 25–27 September 2012, Brisbane, Australia. Australian Centre for Geomechanics, The University of Western Australia. 2012.

[14] Keim JL, Lele SR, DeWitt PD, Fitzpatrick JJ, Jenni NS. Estimating the intensity of use by interacting predators and prey using camera traps. Journal of Animal Ecology. 2019;. 10.1111/1365-2656.12960 30834526

[15] Rooney RC, Bayley SE, Schindler DW. Oil sands mining and reclamation cause massive loss of peatland and stored carbon. Proceedings of the National Academy of Sciences. 2012;109(13):4933–7. 10.1073/pnas.1117693108 PMC3323950 22411786

[16] Pickard D, Hall A, Murray C, Frid L, Schwarz C, Ochoski N. Long-term plot network: effectiveness monitoring program. Prepared for the Plot Network Task Group, Terrestrial Subgroup, Reclamation Working Group, Cumulative Environmental Management Association (CEMA), Fort McMurray, AB. 108 pp. 2013.

[17] Salafsky N, Margoluis R, Redford KH. Adaptive management: A tool for conservation practitioners. Washington, D.C 2001.

[18] Stefani F, Isabel N, Morency M-J, Lamothe M, Nadeau S, Lachance D, et al The impact of reconstructed soils following oil sands exploitation on aspen and its associated belowground microbiome. Scientific Reports. 2018;8(1):2761 10.1038/s41598-018-20783-6 29426844 PMC5807544

[19] Audet P, Pinno BD, Thiffault E. Reclamation of boreal forest after oil sands mining: anticipating novel challenges in novel environments. Canadian Journal of Forest Research. 2014;45(3):364–71.

[20] Allen EW. Process water treatment in Canada’s oil sands industry: Target pollutants and treatment objectives. Journal of Environmental Engineering and Science. 2008;7(2):123–38.

[21] Rogers VV, Wickstrom M, Liber K, MacKinnon MD. Acute and subchronic mammalian toxicity of naphthenic acids from oil sands tailings. Toxicological Sciences. 2002;66(2):347–55. 10.1093/toxsci/66.2.347 11896302

[22] Hammond JHE, Hoffman PGK, Pinno BD, Pinzon J, Klimaszewski J, Hartley DJ. Response of ground and rove beetles (Coleoptera: Carabidae, Staphylinidae) to operational oil sands mine reclamation in northeastern Alberta, a case study. Journal of Insect Conservation. 2018:1–20.

[23] Beckingham JD, Archibald JH. Field guide to ecosites of Northern Alberta. Natural Resources Canada. Canadian Forest Service, Northwest Region, Northern Forestry Centre. Special Report 5. Edmonton Alberta. 1996.

[24] Hawkes VC, Hentze N, McKinnon B, Wood CM. Early successional wildlife monitoring program Canadian Natural Resources Limited Horizon Oil Sands. Year 2 2013–2014 annual report. LGL Report EA3368A. Unpublished report by LGL Limited environmental research associates, Sidney, BC, for Canadian Natural Resources Limited, Fort McMurray, AB. 65 pp + Appendices. 2014.

[25] Hawkes VC, Tuttle KN, McKinnon BG, Hentze N. Early successional wildlife monitoring on reclaimed plots in the oil sands region. 2010–2012 comprehensive report. LGL Report EA3248. Unpublished report by LGL Limited environmental research associates, Sidney, BC, for CEMA–The Reclamation Working Group (RWG), Fort McMurray, AB. 59 pp + Appendices. 2013.

[26] Rowland SM, Prescott CE, Grayston SJ, Quideau SA, Bradfield GE. Recreating a functioning forest soil in reclaimed oil sands in northern Alberta: An approach for measuring success in ecological restoration. Journal of Environmental Quality. 2009;38(4):1580–90. 10.2134/jeq2008.0317 19549934

[27] Williams JW, Jackson ST, Kutzbach JE. Projected distributions of novel and disappearing climates by 2100 AD. Proceedings of the National Academy of Sciences. 2007;104(14):5738–42. 10.1073/pnas.0606292104 PMC1851561 17389402

[28] Choi YD, Temperton VM, Allen EB, Grootjans AP, Halassy M, Hobbs RJ, et al Ecological restoration for future sustainability in a changing environment. Ecoscience. 2008;15(1):53–64.

[29] Hawkes VC, Wood CM, Hentze N, Johnston NN, Challenger W, Roias S. Regional Early Successional Wildlife Dynamics on Reclaimed Habitats in the Athabasca Oil Sands Region Fort McMurray, Alberta. Year 1 2016. Unpublished report by LGL Limited environmental research associates, Sidney, BC, for Canadian Natural Resources Limited, Fort McMurray, AB. 136 pp + Appendices. 2018.

[30] Andersen AN. My indicator or yours? Making the selection? Journal of Insect Conservation. 1999;3:61–4.

[31] Asif N, Malik M, Chaudhry FN. A review of environmental pollution bioindicators. Pollution. 2018;4(1):111–8.

[32] Burger J. Bioindicators: a review of their use in the environmental literature 1970–2005. Environmental Bioindicators. 2006;1(2):136–44.

[33] Chase MK, Geupel GR. The use of avian focal species for conservation planning in California. Bird Conservation Implementation and Integration in the Americas: Proceedings of the Third International Partners in Flight Conference, General Technical Report PSWGTR-191. Albany, CA: USDA Forest Service. 130–142. 2005.

[34] Feinsinger P. Designing field studies for biodiversity conservation: Island Press; 2001.

[35] McGeoch MA. The selection, testing and application of terrestrial insects as bioindicators. Biological Reviews. 1998;73(2):181–201.

[36] Scopel LC, Diamond AW, Kress SW, Hards AR, Shannon P. Seabird diets as bioindicators of Atlantic herring recruitment and stock size: a new tool for ecosystem-based fisheries management. Canadian Journal of Fisheries and Aquatic Sciences. 2017;(999):1–15.

[37] Moreno CE, Sánchez-Rojas G, Pineda E, Escobar F. Shortcuts for biodiversity evaluation: A review of terminology and recommendations for the use of target groups, bioindicators and surrogates. International Journal of Environment and Health. 2007;1(1):71–86.

[38] Robataille R, Proulx G. Early successional wildlife monitoring program design. Unpublished report by TECO Natural Resource Group and Alpha Wildlife Research Management for the Wildlife Task Group (WTG) Of the Cumulative Environmental Management Association (CEMA), Fort McMurray, Alberta. 50 pp + Appendices. 2010.

[39] Best TL. Bats: Biology and behaviour. Journal of Mammalogy. 1997;78(3):986.

[40] Patriquin KJ, Barclay RMR. Foraging by bats in cleared, thinned and unharvested boreal forest. Journal of Applied Ecology. 2003;40(4):646–57.

[41] Mickleburgh SP, Hutson AM, Racey PA. A review of the global conservation status of bats. Oryx. 2002;36(1):18–34.

[42] Carignan V, Villard MA. Selecting indicator species to monitor ecological integrity: a review. Environmental Monitoring and Assessment. 2002;78(1):45–61. 12197640 10.1023/a:1016136723584

[43] Padoa-Schioppa E, Baietto M, Massa R, Bottoni L. Bird communities as bioindicators: The focal species concept in agricultural landscapes. Ecological Indicators. 2006;6(1):83–93.

[44] Jones G, Jacobs DS, Kunz TH, Willig MR, Racey PA. Carpe noctem: The importance of bats as bioindicators. Endangered Species Research. 2009;8(1–2):93–115.

[45] Wasser SK, Keim JL, Taper ML, Lele SR. The influences of wolf predation, habitat loss, and human activity on caribou and moose in the Alberta oil sands. Frontiers in Ecology and the Environment. 2011;9(10):546–51.

[46] Martiniakova M, Omelka R, Jancova A, Formicki G, Stawarz R, Bauerova M. Accumulation of risk elements in kidney, liver, testis, uterus and bone of free-living wild rodents from a polluted area in Slovakia. Journal of Environmental Science and Health. 2012;47(9):1202–6. 10.1080/10934529.2012.672062 22540640

[47] Rainio J, Niemelä J. Ground beetles (Coleoptera: Carabidae) as bioindicators. Biodiversity & Conservation. 2003;12(3):487–506.

[48] Underwood AJ. Experiments in ecology: Their logical design and interpretation using analysis of variance. New York, NY: Cambridge University Press; 1997 504 p.

[49] Underwood AJ, Chapman MG, Connell SD. Observations in ecology: you can’t make progress on processes without understanding the patterns. Journal of Experimental Marine Biology and Ecology. 2000;250(1):97–115. 10969165 10.1016/s0022-0981(00)00181-7

[50] Ralph CJ, Sauer JR, Droege S. Monitoring bird populations by point counts. Gen. Tech. Rep. PSW-GTR-149. Albany, CA: US Department of Agriculture, Forest Service, Pacific Southwest Research Station. 187 p. 1995.

[51] Clarke KR. Non-parametric multivariate analyses of changes in community structure. Australian Journal of Ecology. 1993;18(1):117–43.

[52] Clarke KR, Somerfield PJ, Chapman MG. On resemblance measures for ecological studies, including taxonomic dissimilarities and a zero-adjusted Bray–Curtis coefficient for denuded assemblages. Journal of Experimental Marine Biology and Ecology. 2006;330(1):55–80.

[53] Anderson M, Gorley RN, Clarke RK. Permanova+ for Primer: Guide to software and statistical methods. Plymouth, United Kingdom: PRIMER-E Ltd; 2008.

[54] Clarke KR, Ainsworth M. A method of linking multivariate community structure to environmental variables. Marine Ecology Progress Series. 1993;92:205–19.

[55] Clarke KR, Gorley RN. PRIMER v7: user manual/tutorial 3rd ed. Plymouth, United Kingdom: Primer-E Ltd; 2015.

[56] Burnham KP, Anderson DR. Model selection and multimodel inference: A practical information-theoretic approach. New York: Springer Verlag 2002.

[57] Charchuk C, Bayne EM. Avian community response to understory protection harvesting in the boreal forest of Alberta, Canada. Forest Ecology and Management. 2018;407:9–15.

[58] Pinno BD, Hawkes VC. Temporal trends of ecosystem development on different site types in reclaimed boreal forests. Forests. 2015;6(6):2109–24.

[59] Bergeron Y, Dubue M. Succession in the southern part of the Canadian boreal forest. Vegetatio. 1988;79(1–2):51–63.

[60] Litvak M, Miller S, Wofsy SC, Goulden M. Effect of stand age on whole ecosystem CO2 exchange in the Canadian boreal forest. Journal of Geophysical Research. 2003;108:1–11.

[61] Siitonen J, Martikainen P, Punttila P, Rauh J. Coarse woody debris and stand characteristics in mature managed and old-growth boreal mesic forests in southern Finland. Forest Ecology and Management. 2000;128(3):211–25.

[62] Cogbill CV. Dynamics of the boreal forests of the Laurentian Highlands, Canada. Canadian Journal of Forest Research. 1985;15(1):252–61.

[63] Pezya J-P, Raouxa A, Marmina S, Balayb P, Dauvina J-C. What are the most suitable indices to detect the structural and functional changes of benthic community after a local and short-term disturbance? Ecological Indicators. 2018;91:2320–240.

[64] Chen HYH, Biswas SR, Sobey TM, Brassard BW, Bartels SF. Reclamation strategies for mined forest soils and overstorey drive understorey vegetation. Journal of Applied Ecology. 2018;55(2):926–36.

