# Supplementary material for: Wildlife usage indicates increased similarity between reclaimed upland habitat and mature boreal forest in the Athabasca Oil Sands Region of Alberta, Canada
Source: PLoS One. 2019 Jun 4;14(6):e0217556. doi: 10.1371/journal.pone.0217556 (PMC6548362; doi:10.1371/journal.pone.0217556)
Supplement: S1 Table — BRN: Burned; CLR: Cleared; COMP: Compensation Lake Forest; LOG: Logged; MF: Mature Forest; REC: Reclaimed. Unidentified species were retained in the analysis to better represent the taxonomic diversity observed on site. Inclusion or exclusion of unidentified taxa did not influence the reported trends. (DOCX) [file pone.0217556.s001.docx]

Supplemental Table S1: Presence/absence of species observed in the Alberta Oil Sands Region as part of the Early Successional Wildlife Dynamics Program. BRN: Burned; CLR: Cleared; COMP: Compensation Lake Forest; LOG: Logged; MF: Mature Forest; REC: Reclaimed. Unidentified species were retained in the analysis to better represent the taxonomic diversity observed on site. Inclusion or exclusion of unidentified taxa did not influence the reported trends.

|  |  |  |  |  |  |  |  |
| --- | --- | --- | --- | --- | --- | --- | --- |
| Common Name | Scientific Name | BRN | CLR | COMP | LOG | MF | REC |
| Alder Flycatcher | *Empidonax alnorum* | 1 | 1 | 1 | 1 | 1 | 1 |
| American Black Bear | *Ursus americanus* | 0 | 1 | 1 | 1 | 1 | 1 |
| American Coot | *Fulica americana* | 0 | 0 | 1 | 0 | 0 | 1 |
| American Crow | *Corvus brachyrhynchos* | 1 | 0 | 1 | 0 | 1 | 1 |
| American Goldfinch | *Spinus tristis* | 0 | 0 | 0 | 0 | 0 | 1 |
| American Kestrel | *Falco sparverius* | 1 | 0 | 1 | 1 | 0 | 1 |
| American Marten | *Martes americana* | 1 | 1 | 1 | 1 | 1 | 1 |
| American Mink | *Neovison vison* | 0 | 0 | 0 | 0 | 0 | 1 |
| American Moose | *Alces alces* | 0 | 0 | 1 | 1 | 1 | 1 |
| American Pipit | *Anthus rubescens* | 0 | 0 | 0 | 0 | 0 | 1 |
| American Red Squirrel | *Tamiasciurus hudsonicus* | 0 | 0 | 1 | 0 | 0 | 0 |
| American Redstart | *Setophaga ruticilla* | 0 | 1 | 1 | 0 | 1 | 1 |
| American Robin | *Turdus migratorius* | 1 | 1 | 1 | 1 | 1 | 1 |
| American Three-toed Woodpecker | *Picoides dorsalis* | 1 | 0 | 0 | 1 | 1 | 0 |
| American Wigeon | *Anas americana* | 0 | 0 | 1 | 0 | 0 | 1 |
| Arctic Shrew | *Sorex arcticus* | 0 | 0 | 0 | 0 | 0 | 1 |
| Bald Eagle | *Haliaeetus leucocephalus* | 0 | 0 | 1 | 0 | 0 | 0 |
| Bank Swallow | *Riparia riparia* | 0 | 0 | 0 | 0 | 0 | 1 |
| Barn Swallow | *Hirundo rustica* | 1 | 0 | 0 | 0 | 1 | 1 |
| Bay-breasted Warbler | *Setophaga castanea* | 0 | 1 | 0 | 0 | 1 | 0 |
| Belted Kingfisher | *Setophaga castanea* | 0 | 0 | 1 | 0 | 1 | 0 |
| Black Tern | *Chlidonias niger* | 0 | 0 | 0 | 0 | 0 | 1 |
| Black-and-white Warbler | *Mniotilta varia* | 0 | 0 | 1 | 0 | 1 | 1 |
| Black-backed Woodpecker | *Picoides arcticus* | 1 | 0 | 1 | 0 | 1 | 0 |
| Black-billed Magpie | *Pica hudsonia* | 0 | 0 | 1 | 0 | 1 | 1 |
| Black-capped Chickadee | *Poecile atricapillus* | 1 | 0 | 1 | 1 | 1 | 1 |
| Blackpoll Warbler | *Setophaga striata* | 0 | 0 | 0 | 0 | 0 | 1 |
| Black-throated Green Warbler | *Setophaga virens* | 0 | 0 | 0 | 0 | 1 | 0 |
| Blue Jay | *Cyanocitta cristata* | 0 | 0 | 1 | 1 | 1 | 1 |
| Blue-headed Vireo | *Vireo solitarius* | 0 | 0 | 0 | 1 | 1 | 1 |
| Blue-winged Teal | *Anas discors* | 0 | 0 | 1 | 0 | 0 | 0 |
| Bonaparte's Gull | *Chroicocephalus philadelphia* | 0 | 0 | 1 | 0 | 0 | 0 |
| Boreal Chickadee | *Poecile hudsonicus* | 0 | 0 | 1 | 0 | 1 | 0 |
| Brewer's Blackbird | *Euphagus cyanocephalus* | 1 | 1 | 1 | 0 | 0 | 1 |
| Broad-winged Hawk | *Buteo platypterus* | 0 | 0 | 1 | 0 | 0 | 0 |
| Brown Creeper | *Certhia americana* | 0 | 0 | 0 | 0 | 1 | 0 |
| Brown-headed Cowbird | *Molothrus ater* | 0 | 0 | 0 | 0 | 0 | 1 |
| Bufflehead | *Bucephala albeola* | 0 | 1 | 1 | 0 | 0 | 1 |
| California Gull | *Larus californicus* | 0 | 0 | 1 | 0 | 0 | 0 |
| Canada Goose | *Branta canadensis* | 1 | 0 | 1 | 0 | 1 | 1 |
| Canada Jay | *Perisoreus canadensis* | 1 | 1 | 1 | 1 | 1 | 1 |
| Canada Lynx | *Lynx canadensis* | 0 | 0 | 1 | 1 | 1 | 1 |
| Canada Warbler | *Cardellina canadensis* | 0 | 0 | 1 | 0 | 1 | 1 |
| Canvasback | *Aythya valisineria* | 0 | 0 | 0 | 0 | 0 | 1 |
| Cape May Warbler | *Setophaga tigrina* | 0 | 0 | 0 | 0 | 1 | 1 |
| Cedar Waxwing | *Bombycilla cedrorum* | 1 | 1 | 1 | 1 | 1 | 1 |
| Chipping Sparrow | *Spizella passerina* | 1 | 1 | 1 | 1 | 1 | 1 |
| Clay-colored Sparrow | *Spizella pallida* | 1 | 1 | 1 | 1 | 1 | 1 |
| Cliff Swallow | *Petrochelidon pyrrhonota* | 0 | 0 | 0 | 0 | 0 | 1 |
| Common Goldeneye | *Bucephala clangula* | 0 | 0 | 1 | 0 | 0 | 1 |
| Common Grackle | *Quiscalus quiscula* | 0 | 0 | 1 | 0 | 0 | 1 |
| Common Loon | *Gavia immer* | 1 | 1 | 1 | 1 | 1 | 1 |
| Common Merganser | *Mergus merganser* | 0 | 0 | 1 | 0 | 1 | 0 |
| Common Nighthawk | *Chordeiles minor* | 0 | 0 | 0 | 1 | 1 | 1 |
| Common Raven | *Corvus corax* | 1 | 1 | 1 | 1 | 1 | 1 |
| Common Shrew | *Sorex araneus* | 0 | 0 | 1 | 0 | 1 | 1 |
| Common Yellowthroat | *Geothlypis trichas* | 1 | 1 | 1 | 0 | 1 | 1 |
| Connecticut Warbler | *Oporornis agilis* | 0 | 0 | 1 | 0 | 1 | 0 |
| Coyote | *Canis latrans* | 0 | 0 | 1 | 0 | 1 | 1 |
| Dark-eyed Junco | *Junco hyemalis* | 1 | 1 | 1 | 1 | 1 | 0 |
| Deer Mouse | *Peromyscus maniculatus* | 1 | 1 | 1 | 1 | 1 | 1 |
| Downy Woodpecker | *Picoides pubescens* | 1 | 0 | 0 | 0 | 1 | 0 |
| Dusky Shrew | *Sorex monticolus* | 0 | 0 | 0 | 0 | 0 | 1 |
| Eared Grebe | *Podiceps nigricollis* | 0 | 0 | 1 | 0 | 0 | 0 |
| Eastern Heather Vole | *Phenacomys ungava* | 0 | 0 | 1 | 0 | 1 | 1 |
| Eastern Kingbird | *Tyrannus tyrannus* | 0 | 0 | 0 | 0 | 0 | 1 |
| European Starling | *Sturnus vulgaris* | 0 | 0 | 0 | 0 | 0 | 1 |
| Evening Grosbeak | *Coccothraustes vespertinus* | 0 | 0 | 0 | 0 | 0 | 1 |
| Fisher | *Pekania pennanti* | 0 | 0 | 1 | 0 | 1 | 0 |
| Forster's Tern | *Sterna forsteri* | 0 | 0 | 1 | 0 | 0 | 0 |
| Golden-crowned Kinglet | *Regulus satrapa* | 0 | 0 | 0 | 0 | 1 | 0 |
| Gray Jay | *Perisoreus canadensis* | 0 | 0 | 0 | 0 | 0 | 1 |
| Gray Wolf | *Canis lupus* | 0 | 0 | 1 | 1 | 1 | 1 |
| Great Blue Heron | *Ardea herodias* | 0 | 0 | 1 | 0 | 0 | 0 |
| Greater White-fronted Goose | *Anser albifrons* | 0 | 0 | 1 | 0 | 0 | 0 |
| Greater Yellowlegs | *Tringa melanoleuca* | 1 | 0 | 1 | 0 | 1 | 1 |
| Green-winged Teal | *Anas carolinensis* | 0 | 0 | 1 | 0 | 0 | 1 |
| Hairy Woodpecker | *Leuconotopicus villosus* | 1 | 1 | 1 | 1 | 1 | 1 |
| Hermit Thrush | *Catharus guttatus* | 1 | 1 | 1 | 1 | 1 | 1 |
| Herring Gull | *Larus argentatus* | 0 | 0 | 1 | 0 | 0 | 1 |
| Hooded Merganser | *Lophodytes cucullatus* | 0 | 0 | 1 | 0 | 0 | 0 |
| Horned Grebe | *Podiceps auritus* | 0 | 0 | 1 | 0 | 0 | 1 |
| Horned Lark | *Eremophila alpestris* | 0 | 0 | 0 | 0 | 0 | 1 |
| Killdeer | *Charadrius vociferus* | 0 | 0 | 1 | 0 | 1 | 1 |
| Lapland Longspur | *Calcarius lapponicus* | 0 | 0 | 0 | 0 | 0 | 1 |
| Least Chipmunk | *Tamias minimus* | 1 | 0 | 0 | 1 | 0 | 1 |
| Least Flycatcher | *Empidonax minimus* | 1 | 1 | 1 | 1 | 1 | 1 |
| Least Weasel | *Mustela nivalis* | 0 | 0 | 1 | 0 | 0 | 1 |
| LeConte's Sparrow | *Ammodramus leconteii* | 1 | 1 | 1 | 0 | 0 | 1 |
| Lesser Scaup | *Aythya affinis* | 0 | 0 | 1 | 0 | 0 | 0 |
| Lesser Yellowlegs | *Tringa flavipes* | 1 | 1 | 1 | 0 | 0 | 1 |
| Lincoln's Sparrow | *Melospiza lincolnii* | 1 | 1 | 1 | 1 | 1 | 1 |
| Long-eared Owl | *Asio otus* | 0 | 0 | 0 | 0 | 0 | 1 |
| Longspur sp. | *Calcarius* sp. | 0 | 0 | 0 | 0 | 0 | 1 |
| Magnolia Warbler | *Setophaga magnolia* | 1 | 0 | 1 | 0 | 1 | 1 |
| Mallard | *Anas platyrhynchos* | 0 | 1 | 1 | 0 | 0 | 1 |
| Marsh Wren | *Cistothorus palustris* | 1 | 0 | 1 | 0 | 0 | 1 |
| Meadow Jumping Mouse | *Zapus hudsonius* | 0 | 0 | 1 | 0 | 0 | 1 |
| Meadow Vole | *Microtus pennsylvanicus* | 1 | 1 | 1 | 1 | 1 | 1 |
| Merlin | *Falco columbarius* | 0 | 0 | 0 | 0 | 0 | 1 |
| Mountain Bluebird | *Sialia currucoides* | 0 | 0 | 0 | 1 | 0 | 0 |
| Mourning Warbler | *Geothlypis philadelphia* | 1 | 1 | 1 | 0 | 1 | 1 |
| Mule Deer | *Odocoileus hemionus* | 0 | 0 | 0 | 0 | 0 | 1 |
| Nashville Warbler | *Leiothlypis ruficapilla* | 0 | 0 | 0 | 0 | 1 | 0 |
| North American Beaver | *Castor canadensis* | 0 | 0 | 1 | 0 | 0 | 0 |
| North American River Otter | *Lontra canadensis* | 0 | 0 | 1 | 0 | 0 | 0 |
| Northern Flicker | *Colaptes auratus* | 1 | 1 | 1 | 1 | 1 | 1 |
| Northern Flying Squirrel | *Glaucomys sabrinus* | 0 | 0 | 0 | 0 | 1 | 0 |
| Northern Harrier | *Circus cyaneus* | 0 | 1 | 1 | 0 | 0 | 1 |
| Northern Hawk Owl | *Surnia ulula* | 1 | 0 | 0 | 0 | 0 | 0 |
| Northern Pintail | *Anas acuta* | 0 | 0 | 1 | 0 | 0 | 0 |
| Northern Shoveler | *Anas clypeata* | 0 | 0 | 1 | 0 | 0 | 1 |
| Northern Waterthrush | *Parkesia noveboracensis* | 0 | 0 | 1 | 0 | 1 | 1 |
| Olive-sided Flycatcher | *Contopus cooperi* | 1 | 0 | 0 | 1 | 0 | 0 |
| Orange-crowned Warbler | *Vermivora celata* | 0 | 0 | 1 | 1 | 0 | 1 |
| Ovenbird | *Seiurus aurocapilla* | 1 | 1 | 1 | 0 | 1 | 1 |
| Palm Warbler | *Setophaga palmarum* | 1 | 0 | 1 | 1 | 0 | 1 |
| Philadelphia Vireo | *Vireo philadelphicus* | 0 | 0 | 1 | 0 | 1 | 1 |
| Pied-billed Grebe | *Podilymbus podiceps* | 0 | 0 | 0 | 0 | 0 | 1 |
| Pileated Woodpecker | *Dryocopus pileatus* | 1 | 1 | 1 | 0 | 1 | 1 |
| Pine Siskin | *Spinus pinus* | 1 | 1 | 1 | 1 | 1 | 1 |
| Purple Finch | *Haemorhous purpureus* | 1 | 1 | 1 | 0 | 1 | 1 |
| Pygmy Shrew | *Sorex minutus* | 0 | 0 | 0 | 0 | 0 | 1 |
| Red Crossbill | *Loxia curvirostra* | 1 | 0 | 0 | 1 | 0 | 0 |
| Red Fox | *Vulpes vulpes* | 0 | 0 | 1 | 1 | 1 | 1 |
| Red Squirrel | *Sciurus vulgaris* | 1 | 1 | 0 | 1 | 1 | 1 |
| Red-breasted Nuthatch | *Sitta canadensis* | 0 | 0 | 0 | 0 | 1 | 1 |
| Red-eyed Vireo | *Vireo olivaceus* | 1 | 1 | 1 | 1 | 1 | 1 |
| Redhead | *Aythya americana* | 0 | 0 | 1 | 0 | 0 | 0 |
| Red-necked Grebe | *Podiceps grisegena* | 0 | 0 | 0 | 1 | 0 | 1 |
| Red-tailed Hawk | *Buteo jamaicensis* | 0 | 0 | 0 | 1 | 0 | 0 |
| Red-winged Blackbird | *Agelaius phoeniceus* | 1 | 0 | 1 | 0 | 1 | 1 |
| Ring-billed Gull | *Larus delawarensis* | 0 | 0 | 0 | 0 | 0 | 1 |
| Ring-necked Duck | *Aythya collaris* | 0 | 0 | 0 | 0 | 0 | 1 |
| Rose-breasted Grosbeak | *Pheucticus ludovicianus* | 1 | 1 | 1 | 0 | 1 | 1 |
| Ruby-crowned Kinglet | *Regulus calendula* | 1 | 0 | 1 | 0 | 1 | 0 |
| Ruddy Duck | *Oxyura jamaicensis* | 0 | 0 | 1 | 0 | 0 | 0 |
| Ruffed Grouse | *Bonasa umbellus* | 0 | 0 | 1 | 0 | 1 | 0 |
| Rusty Blackbird | *Euphagus carolinus* | 0 | 0 | 1 | 0 | 0 | 1 |
| Sandhill Crane | *Grus canadensis* | 1 | 1 | 1 | 1 | 1 | 1 |
| Savannah Sparrow | *Passerculus sandwichensis* | 1 | 1 | 1 | 0 | 0 | 1 |
| Sedge Wren | *Cistothorus stellaris* | 0 | 0 | 0 | 0 | 0 | 1 |
| Sharp-shinned Hawk | *Accipiter striatus* | 0 | 0 | 1 | 0 | 1 | 0 |
| Sharp-tailed Grouse | *Tympanuchus phasianellus* | 1 | 0 | 1 | 1 | 0 | 1 |
| Short-tailed Weasel | *Mustela erminea* | 1 | 0 | 1 | 0 | 1 | 1 |
| Smith's Longspur | *Calcarius pictus* | 0 | 0 | 0 | 0 | 0 | 1 |
| Snowshoe Hare | *Lepus americanus* | 0 | 1 | 1 | 0 | 1 | 1 |
| Solitary Sandpiper | *Tringa solitaria* | 1 | 0 | 0 | 0 | 0 | 1 |
| Song Sparrow | *Melospiza melodia* | 1 | 1 | 1 | 0 | 1 | 1 |
| Sora | *Porzana carolina* | 1 | 1 | 1 | 0 | 0 | 1 |
| Sorex sp. | *Sorex* sp. | 1 | 1 | 1 | 0 | 1 | 1 |
| Southern Red-backed Vole | *Myodes gapperi* | 1 | 1 | 1 | 1 | 1 | 1 |
| Spotted Sandpiper | *Actitis macularius* | 0 | 0 | 1 | 0 | 1 | 1 |
| Swainson's Thrush | *Catharus ustulatus* | 1 | 1 | 1 | 0 | 1 | 1 |
| Swamp Sparrow | *Melospiza georgiana* | 1 | 1 | 1 | 0 | 1 | 1 |
| Tennessee Warbler | *Leiothlypis peregrina* | 1 | 1 | 1 | 1 | 1 | 1 |
| Tree Swallow | *Tachycineta bicolor* | 1 | 0 | 1 | 1 | 0 | 1 |
| Tundra Swan | *Cygnus columbianus* | 1 | 0 | 0 | 0 | 0 | 0 |
| Unidentified Flycatcher | - | 0 | 0 | 0 | 1 | 0 | 0 |
| Unidentified Larus Gull | - | 0 | 0 | 0 | 0 | 0 | 1 |
| Unidentified Scaup | - | 0 | 0 | 1 | 0 | 0 | 0 |
| Vesper Sparrow | *Pooecetes gramineus* | 0 | 1 | 1 | 1 | 0 | 1 |
| Warbling Vireo | *Vireo gilvus* | 0 | 0 | 1 | 0 | 1 | 0 |
| Western Heather Vole | *Phenacomys intermedius* | 1 | 0 | 0 | 0 | 1 | 0 |
| Western Tanager | *Piranga ludoviciana* | 1 | 1 | 1 | 1 | 1 | 1 |
| Western Wood-Pewee | *Contopus sordidulus* | 1 | 1 | 1 | 1 | 1 | 0 |
| White-crowned Sparrow | *Zonotrichia leucophrys* | 0 | 0 | 0 | 0 | 0 | 1 |
| White-tailed Deer | *Odocoileus virginianus* | 0 | 1 | 1 | 1 | 1 | 1 |
| Deer spp. | - | 0 | 0 | 1 | 0 | 1 | 1 |
| White-throated Sparrow | *Zonotrichia albicollis* | 1 | 1 | 1 | 1 | 1 | 1 |
| White-winged Crossbill | *Loxia leucoptera* | 1 | 0 | 1 | 1 | 1 | 1 |
| Wilson's Snipe | *Gallinago delicata* | 1 | 1 | 1 | 0 | 1 | 1 |
| Wilson's Warbler | *Cardellina pusilla* | 1 | 1 | 1 | 1 | 1 | 1 |
| Winter Wren | *Troglodytes hiemalis* | 0 | 0 | 1 | 0 | 1 | 1 |
| Wolverine | *Gulo gulo* | 0 | 0 | 1 | 0 | 1 | 0 |
| Wood Frog | *Lithobates sylvaticus* | 0 | 0 | 1 | 0 | 0 | 1 |
| Woodchuck | *Marmota monax* | 0 | 0 | 1 | 0 | 0 | 0 |
| Yellow Warbler | *Setophaga petechia* | 1 | 1 | 1 | 1 | 1 | 1 |
| Yellow-bellied Flycatcher | *Empidonax flaviventris* | 1 | 0 | 1 | 0 | 0 | 0 |
| Yellow-bellied Sapsucker | *Sphyrapicus varius* | 1 | 1 | 1 | 0 | 1 | 1 |
| Yellow-headed Blackbird | *Xanthocephalus xanthocephalus* | 0 | 0 | 0 | 0 | 0 | 1 |
| Yellow-rumped Warbler | *Setophaga coronata* | 1 | 1 | 1 | 1 | 1 | 1 |
|  |  |  |  |  |  |  |  |
