# Supplementary material for: Wildlife usage indicates increased similarity between reclaimed upland habitat and mature boreal forest in the Athabasca Oil Sands Region of Alberta, Canada
Source: PLoS One. 2019 Jun 4;14(6):e0217556. doi: 10.1371/journal.pone.0217556 (PMC6548362; doi:10.1371/journal.pone.0217556)
Supplement: S3 Table — (DOCX) [file pone.0217556.s003.docx]

Table S3: Comparison of linear, quadradic, and cubic models comparing community similarity against time since reclamation (age) in the Athabasca Oil Sands Region.

|  |  |  |  |  |  |
| --- | --- | --- | --- | --- | --- |
| Model | *p* | R^2^ | AIC | ΔAIC | AIC*w* |
| Cubic | 0.0001 | 16 | 5340.68 | 0 | 1 |
| Quadradic | 0.0001 | 15 | 5354.66 | 14 | 0 |
| Linear | 0.0001 | 15 | 5355.05 | 14 | 0 |
|  |  |  |  |  |  |
